# Persistence of Hepatitis B Virus DNA and the Tempos between Virion Secretion and Genome Maturation in a Mouse Model

**DOI:** 10.1128/JVI.01001-19

**Published:** 2019-10-29

**Authors:** Szu-Yao Wu, Ya-Shu Chang, Tien-Hua Chu, Chiaho Shih

**Affiliations:** a Program in Molecular Medicine, National Yang-Ming University and Academia Sinica, Taipei, Taiwan; b Institute of Biomedical Sciences, Academia Sinica, Taipei, Taiwan; c Institute of Microbiology and Immunology, National Yang-Ming University, Taipei, Taiwan; University of Southern California

**Keywords:** hepatitis B virus, HBV, naturally occurring mutation, HBV core antigen, immature virion secretion, genome maturation, hydrodynamic mouse model, HBV core protein, mouse model, naturally occurring variants, virion secretion

## Abstract

Hepatitis B virus (HBV) core protein (HBc) accumulates frequent mutations in natural infection. Wild-type HBV is known to secrete predominantly virions containing mature DNA genome. However, a frequent naturally occurring HBc variant, I97L, changing from an isoleucine to a leucine at amino acid 97, exhibited an immature secretion phenotype in culture, which preferentially secretes virions containing immature genomes. In contrast, mutant P130T, changing from a proline to a threonine at amino acid 130, exhibited a hypermaturation phenotype by accumulating an excessive amount of intracellular fully mature DNA genome. Using a hydrodynamic delivery mouse model, we studied the *in vivo* behaviors of these two mutants, I97L and P130T. We detected no naked core particles in all hydrodynamically injected mice. Mutant I97L in mice exhibited pleiotropic phenotypes: (i) excessive numbers of serum HBV virions containing immature genomes, (ii) significantly reduced numbers of intracellular relaxed-circle and single-stranded DNAs, and (iii) less persistent intrahepatic and secreted HBV DNAs than wild-type HBV. These pleiotropic phenotypes were observed in both immunocompetent and immunodeficient mice. Although mutant P130T also displayed a hypermaturation phenotype *in vivo*, it cannot efficiently rescue the immature virion secretion of mutant I97L. Unexpectedly, the single mutant P130T exhibited *in vivo* a novel phenotype in prolonging the persistence of HBV genome in hepatocytes. Taken together, our studies provide a plausible rationale for HBV to regulate envelopment morphogenesis and virion secretion via genome maturity, which is likely to play an important role in the persistence of viral DNA in this mouse model.

**IMPORTANCE** Chronic infection with human hepatitis B virus (HBV) could lead to cirrhosis and hepatoma. At present, there is no effective treatment to eradicate the virus from patients. HBV in chronic carriers does not exist as a single homogeneous population. The most frequent naturally occurring mutation in HBV core protein occurs at amino acid 97, changing an isoleucine to leucine (I97L). One dogma in the field is that only virions containing a mature genome are preferentially secreted into the medium. Here, we demonstrated that mutant I97L can secrete immature genome in mice. Although viral DNA of mutant I97L with immature genome is less persistent than wild-type HBV in time course experiments, viral DNA of mutant P130T with genome hypermaturation, surprisingly, is more persistent. Therefore, virion secretion regulated by genome maturity could influence viral persistence. It remains an open issue whether virion secretion could be a drug target for HBV therapy.

## Abstract (precis)

Chronic infection with human hepatitis B virus (HBV) could lead to cirrhosis and hepatoma. At present, there is no effective treatment to eradicate the virus from patients. HBV in chronic carriers does not exist as a single homogeneous population. The most frequent naturally occurring mutation in HBV core protein occurs at amino acid 97, changing an isoleucine to leucine (I97L). One dogma in the field is that only virions containing a mature genome are preferentially secreted into the medium. Here, we demonstrated that mutant I97L can secrete immature genome in mice. Although viral DNA of mutant I97L with immature genome is less persistent than wild-type HBV in time course experiments, viral DNA of mutant P130T with genome hypermaturation, surprisingly, is more persistent. Therefore, virion secretion regulated by genome maturity could influence viral persistence. It remains an open issue whether virion secretion could be a drug target for HBV therapy.

## INTRODUCTION

Hepatitis B virus (HBV) is a human hepatotropic DNA virus (hepadnavirus) (1, 2). There are at least 300 million HBV chronic carriers worldwide, and these carriers have a higher risk of developing liver cirrhosis, liver failure, and hepatocellular carcinoma (3). At present, no treatment can effectively eradicate the virus from chronic patients (4). In many patients, lifetime treatment is needed to continuously suppress HBV reactivation. Some HBV patients are coinfected with hepatitis delta virus, leading to more severe liver diseases (5). HBV encodes a core protein (HBc) for nucleocapsid assembly. Additional functions of HBc include its interactions with the pregenomic RNA (pgRNA) and HBV polymerase during pgRNA encapsidation (6). HBV could regulate RNA encapsidation and DNA synthesis by maintaining electrostatic homeostasis within the capsid interior via HBc phosphorylation and dephosphorylation (7–10). HBc is capable of shuttling between nucleus and cytoplasm, because it contains both nuclear localization signal and nuclear export signal (11–13). Overall, HBc is an important multifunctional protein in the HBV life cycle (6).

**FIG 1:**
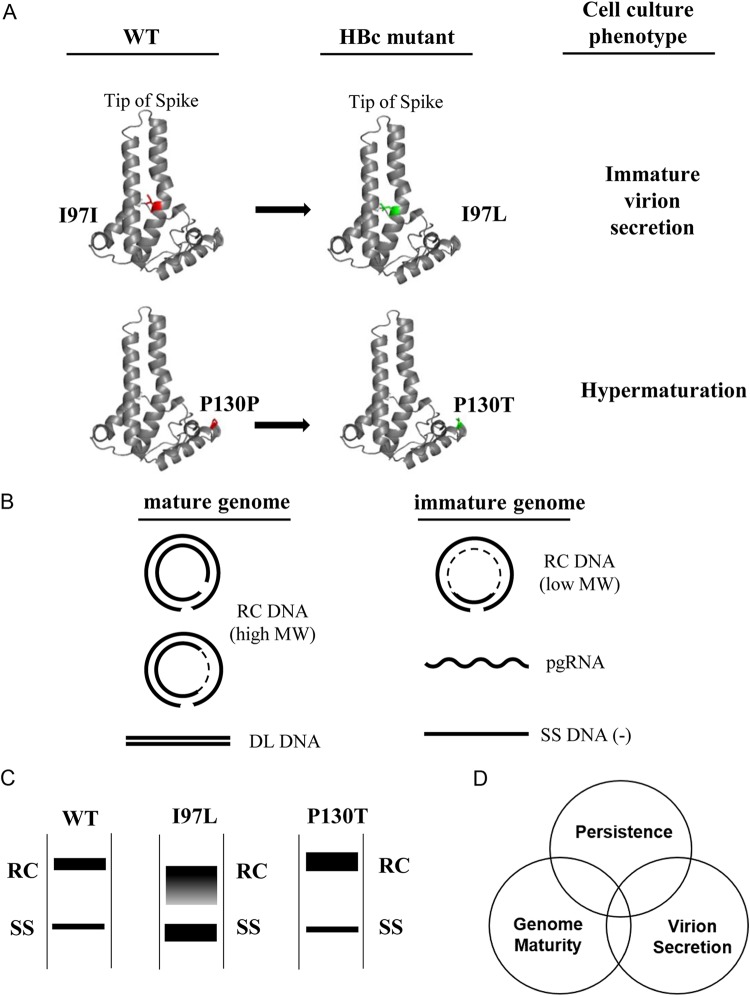
HBV core variants I97L and P130T exhibited abnormal behaviors in viral DNA synthesis and virion secretion. (A) Two frequent natural mutations of HBV core (HBc) antigen occur at amino acids 97 and 130 in chronic hepatitis B patients. The image is a schematic representation of the fold of the HBV capsid protein monomer based on the published crystal structure (PDB code 1QGT) by use of the PyMOL program. Red, wild-type amino acid; green, HBc mutants. Cell culture phenotypes are as explained below for panels B and C. (B) Illustrations of mature and immature HBV genomes. pgRNA, pregenomic RNA; SS DNA, single-stranded (–) DNA reversed transcribed from pgRNA (+); RC DNA, partially double-stranded relaxed circle DNA. DL, low-abundance double-stranded linear DNA, which can be separated from RC DNA after a longer period of electrophoresis. A dotted line of RC molecules represents the single-strand gap region with a variable size in an HBV population. (C) Different Southern blot patterns of virion-associated HBV DNA genomes from the wild type and HBc mutants. (D) Diagram of the trilateral relationships among persistence, genome maturity, and virion secretion.

HBc is known to be a major target of cytotoxic T lymphocytes (14). HBc sequences were compared between asymptomatic carriers and patients with chronic liver diseases (15, 16). Frequent HBc mutations occurred in HBc peptide 84-101 in patients with hepatocellular injury and active hepatitis. The most common HBc variant contains a substitution mutation at amino acid 97, changing an isoleucine to leucine (I97L) (Fig. 1A) (15). Outside this mutation clustering region (HBc 84-101), new hot spot missense mutations at amino acids 5, 60, and 130 were also found in patient samples (17, 18). These missense mutations and other less frequent HBc internal deletions coincide with mapped T-cell epitopes and are thought to influence viral persistence and disease severity (19–23). Acute exacerbations of chronic hepatitis B are associated with increased T-cell responses to hepatitis B core and e antigens (24). The emergence of HBc variants can be detected around the time of acute exacerbation or immune clearance.

Wild-type hepadnavirus is known to preferentially secrete virions containing predominantly mature genome of partially double-stranded (ds) relaxed circle (RC) and ds linear DNAs (Fig. 1B). This phenomenon implied a regulated morphogenesis process whereby genome maturation within nucleocapsids is somehow tightly coupled to envelopment before virion secretion (25). It was envisaged that genome maturation could induce a conformational change in the nucleocapsid (a genome maturation signal), which in turn can facilitate the interaction between nucleocapsids and HBV surface proteins leading to virion secretion. However, in addition to genome-containing virions, HBV also secretes genome-free empty virions (26–31). A revised model proposed that encapsidated single-strand (SS) nucleic acids (SS RNA or SS DNA) constitutes an inhibitory signal for envelopment and virion secretion (28). Without such an inhibitory signal, empty capsids and mature capsids containing dsDNA are free for envelopment and virion secretion. All of these models predict that capsids containing immature genome (SS DNA and SS RNA) are selected against envelopment and secretion.

In contrast to these model predictions, HBc mutant 97L exhibited an “immature secretion phenotype,” which is characterized by nonselective and excessive secretion of virion particles containing immature genomes of SS DNA and low-molecular-weight (low-MW) RC DNA (Fig. 1B and C) (32–34). While HBc mutant I97L is frequent in the genotype C (serotype *adr*), mutant F97L is common in the genotype D (serotype *ayw*) (35; see also references cited in reference 32). Using a *cis-trans* genetic test design, we demonstrated that it is the *trans*-acting mutant F97L HBc protein, rather than the mutant F97L pgRNA, that is responsible for the extracellular immature virion secretion (32). In addition to the *trans*-defect in virion secretion, a *cis*-defect in intracellular viral DNA synthesis was also detected in mutant F97L by a genetic complementation assay (32, 36). Near amino acid 97, an engineered HBc mutation L95A or K96A, blocked envelopment and virion secretion (37). Of note, an immature secretion-like phenomenon was also found in woodchuck and snow goose hepadnaviruses (38–40). Furthermore, immature secretion is not limited to the release of virion-associated SS DNA. Recently, RNA-containing virions were also found in the blood samples of patients (41–43). It remains an open research area whether serum HBV RNA can be used as a reliable marker for monitoring the therapeutic efficacy and the intrahepatic covalently closed circular DNA (cccDNA) level (44–47).

In addition to this HBc 97L mutation, HBc P130T and P5T mutations were found frequently in patients. A proline (P) residue at amino acids 5 and 130 is highly evolutionarily conserved even in woodchuck hepatitis B virus core protein (17). Both P130T and P5T could compensate for or rescue the immature secretion phenotype of mutant I97L (48, 49). Interestingly, while a single HBc mutation L60V or P5T displayed a low-level secretion phenotype (50), a single mutation P130T led to a “hypermaturation phenotype” characterized by an increased abundance of both intracellular and extracellular fully mature full-length RC DNA (48) (Fig. 1). In general, it is believed that the core-envelope interaction is critical for the structural regulation of virion secretion. Point mutation A119F in the pre-S1 region of the envelope protein can compensate for the immature secretion of mutant I97L (51). Using a bioinformatics and genetic approach, we previously identified a hydrophobic pocket around HBc amino acid 97, which could be involved in the putative signal transduction of virion secretion (52). Cryo-electron microscopy (cryoEM) studies revealed significant structural differences in this hydrophobic pocket between the RNA- and DNA-containing capsids (53). Recently, in another cryoEM study, this pocket in the mutant F97L virions was found enlarged (54).

So far, these naturally occurring HBc mutants had only been characterized in tissue culture. In this study, we investigated HBc variants *in vivo* by a hydrodynamic delivery mouse model. The immature secretion of HBc variant I97L can be fully recapitulated *in vivo*. In both immunocompetent BALB/c and immunodeficient IFNAR^−/−^, STAT1^−/−^, and NOD/SCID mouse models, intracellular HBV DNA of mutant I97L is less abundant and more transient than the wild-type HBV. In contrast, mutant P130T exhibited a hypermaturation phenotype with accumulated mature RC form DNA in the mouse liver. Strikingly, relative to wild-type HBV, single mutant P130T significantly prolonged the persistence of intracellular HBV DNA genome. In the double mutant I97L/P130T, mutation P130T can only partially rescue the immature secretion of mutant I97L. In summary, virion secretion regulated by genome maturity could influence viral persistence. These intricate relationships between HBV genome maturation, virion secretion, and persistence were dissected and discussed (Fig. 1D).

## RESULTS

As described in the introduction, we observed previously an immature secretion phenotype of the HBc variant I97L in the tissue culture system (32, 33). To investigate the immature secretion phenotype of mutant I97L in an *in vivo* experimental setting, we introduced HBV DNA (*adr*) of wild-type (WT) and mutant I97L into BALB/c mouse liver by hydrodynamic delivery. At 3 days postinjection (dpi), intracellular core particle-associated DNAs were extracted from mouse liver, and extracellular viral particles were prepared from the pooled mouse sera before Southern blot analysis.

### Mutant I97L secreted more immature HBV genomes than its parental wild-type HBV in BALB/c mice.

**FIG 2:**
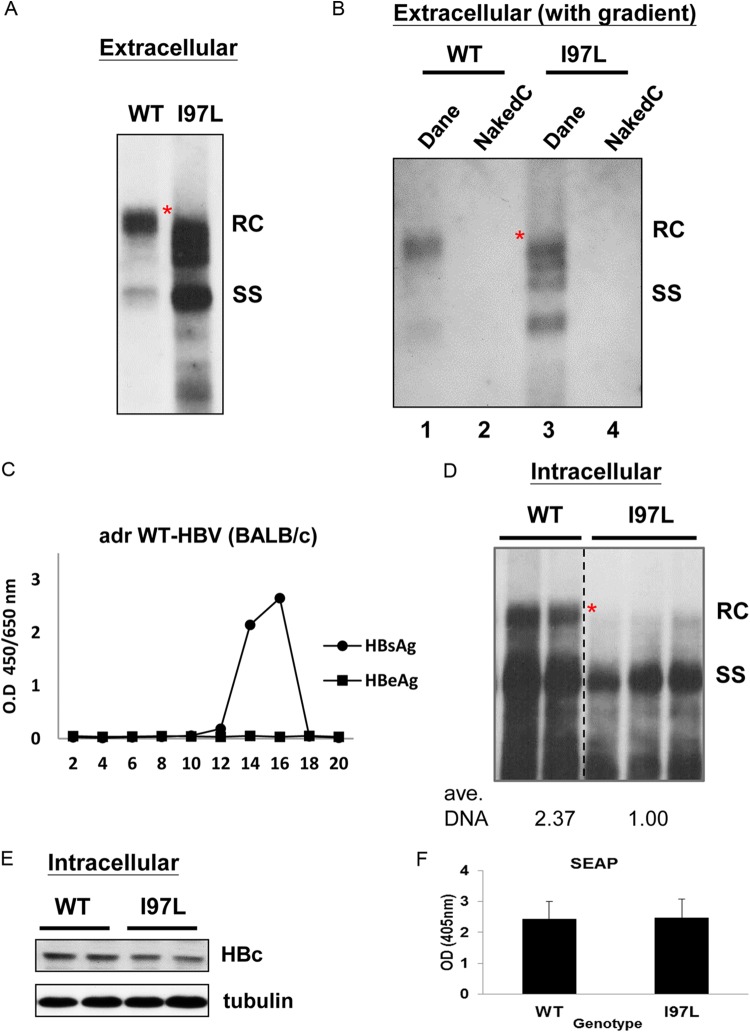
HBc mutant I97L exhibited an immature secretion phenotype by *in vivo* hydrodynamic delivery. BALB/c mice were hydrodynamically injected with 30 μg of plasmid DNAs of a WT HBV (*adr*) or mutant I97L. At 3 dpi, the mice were sacrificed, and both liver and serum samples were collected for assays of HBV replication and virion secretion. (A) Extracellular HBV DNAs were prepared directly from equal volumes of mouse sera of WT and mutant I97L injections before Southern blot analysis. An immature secretion phenotype of HBc mutant I97L in tissue culture was recapitulated in this *in vivo* experimental setting. The red asterisk highlights the lessened abundance of fully mature full-length RC DNA associated with mutant I97L. (B) Purified HBV particles in mouse sera were pooled from fractions 9 to 12 by gradient centrifugation (see Materials and Methods) before Southern blot analysis. Unlike the WT, mutant I97L displayed an excessive number of immature genomes. Dane, Dane particles refer to enveloped virions. NakedC, nonenveloped naked core particles were not detected in the higher-density fractions (see the text). The results here represent one of two independent repeat experiments. (C) No naked core particles can be detected in serum samples of wild-type HBV DNA-injected BALB/c mice. Serum samples were subjected to cesium chloride gradient centrifugation, and HBsAg-positive fractions were detected by HBsAg ELISA (see Materials and Methods). No positive signal was detected in any fractions by HBeAg ELISA. (D) Intracellular core-associated HBV DNA was extracted from the liver tissue and subjected to Southern blotting. The full-length RC form was almost undetectable in mutant I97L. Each lane here represents different DNA samples from each block of approximately 100 mg of liver mass dissected from each injected mouse. This same protocol was used in most of the experiments in other figures. The dotted vertical line indicates splicing from the same gel. The results here represent one of three independent repeat experiments. The amounts of total DNA were quantified by measuring the intensities of full-length RC and full-length SS DNAs using densitometry and ImageJ software. The averaged total DNAs are calculated from two mice injected with WT HBV DNA and normalized to the averaged value from three mice injected with mutant I97L. (E) Detection of only slightly reduced amounts of HBV core protein in the liver lysates of mutant I97L by Western blotting. Each lane represents one liver sample from one injected mouse. (F) Plasmid SEAP encoding a secretable alkaline phosphatase was coinjected with an HBV tandem dimer in panel A. The SEAP activities in the sera indicated similar transfection efficiencies between WT and I97L in hydrodynamic delivery.

As shown in Fig. 2A, mutant I97L released into blood circulation an excessive amount of immature HBV genomes, including both lower-MW RC and SS viral DNA. A closer examination of the Southern blot profiles of HBV replicative intermediates revealed reproducibly the lack or reduction of the full-length RC form in mutant I97L (highlighted by a red asterisk in Fig. 2A). It is well known that HBV in cell culture can secrete so-called naked core particles without an envelope (55). These particles in the medium contain predominantly immature genome of SS DNA. To exclude the possibility that the immature genomes of I97L HBV in Fig. 2A was from the contamination of naked core particles, we separated the lower-density fractions of (enveloped) virions from the higher-density fractions of naked (unenveloped) core particles by CsCl density gradient centrifugation analysis (Fig. 2B). Fractions 9 to 12, with densities around 1.24 g/cm^3^, were pooled for virion-associated viral DNA extraction. By Southern blotting, we detected an abundant amount of immature viral genomes in the virion fractions of mutant I97L, but not in WT HBV (compare lanes 1 and 3 in Fig. 2B). The lack of fully matured full-length RC form in mutant I97L is highlighted by a red asterisk in Fig. 2B. By Southern blotting (lanes 2 and 4, Fig. 2B), we detected no naked core particles (1.35 g/cm^3^) in the sera in all gradient fractions from both WT and mutant I97L injected BALB/c mice. Similarly, we detected no naked core particles in the sera in all gradient fractions from wild-type HBV DNA-injected BALB/c mice by HBeAg enzyme-linked immunosorbent assay (ELISA) (Fig. 2C). Taken together, the immature virion secretion of mutant I97L is not due to the contamination from naked core particles *in vivo*, since they were not detected here.

### Mutant I97L exhibited a reduced level of intracellular relaxed circular DNA.

To examine the intracellular viral DNA replication between WT and mutant I97L in the injected mouse liver, we extracted viral DNA from the freshly dissected liver and performed Southern blot analysis (100 mg liver mass/lane, Fig. 2D). Previously, we observed no apparent deficiency of intracellular viral DNA replication of mutant I97L in cell culture (34). Here, in the *in vivo* setting in BALB/c mice, overall reduction by at least 2.37-fold in I97L total viral DNA synthesis (measurements of full-length RC plus full-length SS DNAs) was observed, with the most striking contrast in the high-MW RC DNA between WT and mutant I97L (Fig. 2D). Again, as highlighted by a red asterisk, fully mature full-length RC form in mutant I97L appeared to be greatly diminished.

To compare the *in vivo* expressions of viral proteins between WT and mutant I97L, we performed Western blot analysis using liver samples from mice sacrificed on 3 dpi. Our results found only marginal difference in HBc expression between WT and mutant I97L (Fig. 2E). To monitor the transfection efficiency of hydrodynamic delivery, we coinjected WT or I97L plasmids with a control plasmid encoding secretable alkaline phosphatase (SEAP). As shown in Fig. 2F, the averaged transfection efficiencies (SEAP activities) between WT and I97L experimental groups were comparable to each other.

### More rapid clearance of intracellular mutant I97L DNA.

**FIG 3:**
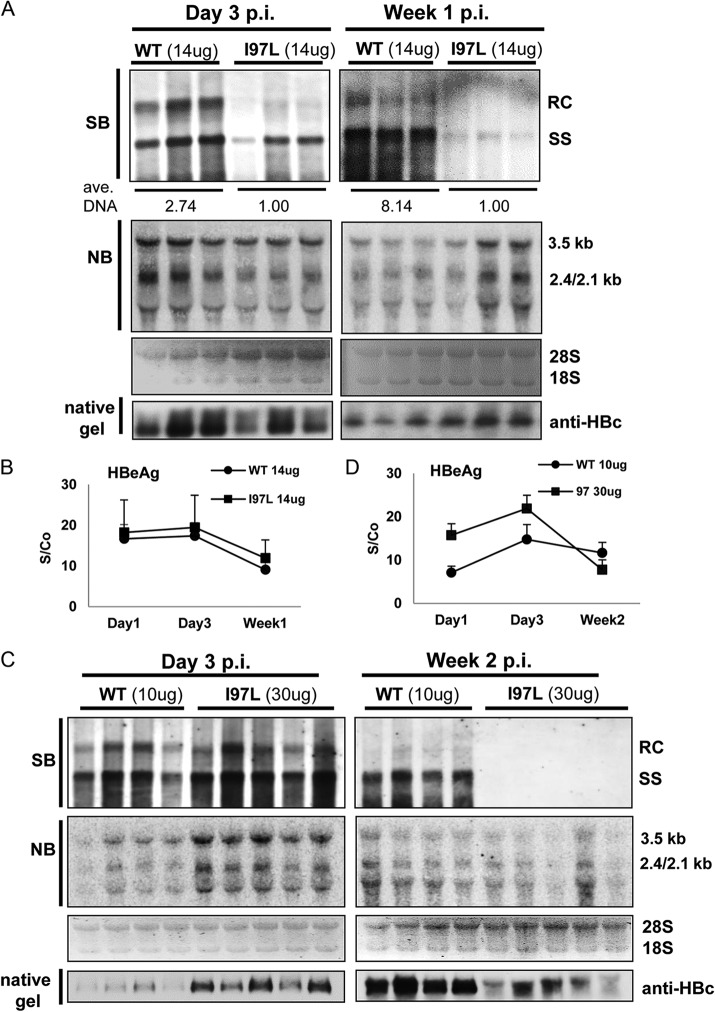
Reduction in the persistence of intracellular HBV DNA level of mutant I97L in the mouse liver by hydrodynamic delivery. (A) Liver samples were collected at 3 days or 1 week after the hydrodynamic injection of 14 μg of HBV plasmid DNAs of WT or mutant I97L. The intrahepatic levels of viral DNA and RNA were determined using Southern and Northern blot analyses. The averaged amounts of total viral DNA were measured and calculated as described in Fig. 2D. Some differences in the intensities of viral RNAs are not representative and are likely due to experimental variations from individual littermate. The viral DNA level of mutant I97L appeared to be much lower than those of WT HBV at day 3 or week 1 postinjection. The intrahepatic levels of ribosomal 28S and 18S RNAs were included as the sample loading control for Northern blotting. The intrahepatic levels of capsid particles were determined by native agarose gel electrophoresis and subsequent immunoblotting with anti-HBc antibodies. The results here represent one of four independent repeat experiments. (B) The serum HBeAg titers in mice receiving WT (●) or I97L (■) DNAs were determined at the indicated time points with an enzyme immunoassay (see Materials and Methods). S/Co, signal/cutoff. Positivity for HBeAg was defined as an S/Co value of ≥ 1. (C) More rapid decay of viral DNA of mutant I97L in hepatocytes was observed by hydrodynamic delivery. As described above, liver samples were collected for Southern and Northern blot analysis at 3 days or 2 weeks after the hydrodynamic injections of 10 μg of WT or 30 μg of mutant I97L HBV DNA. Although the viral DNA levels were comparable between 10 μg of WT and 30 μg of I97L at 3 dpi (left panel), no viral DNA signal of I97L was detectable at 2 weeks postinjection (right panel). (D) Detection of extracellular levels of HBeAg was as described above.

The results in Fig. 2D showed a decreased level of intracellular viral DNA of mutant I97L at day 3 postinjection. We compared the intracellular levels of viral DNAs in livers between WT and mutant I97L at 1 week postinjection (Fig. 3A). At 3 dpi with 14 μg of HBV plasmid DNA (left panel), DNA replicative intermediates of mutant I97L were already weaker than those of WT, while the cytoplasmic viral RNA and intrahepatic capsid particles were comparable between WT and mutant I97L on the agarose gels. At 1 week postinjection (right panel), the DNA level of mutant I97L became further reduced than that of WT HBV as determined by Southern blotting, despite the fact that mutant I97L displayed stronger signals of viral RNA, capsid particles, and serum HBeAg (Fig. 3A and B). The ratio of the averaged signal intensities of total viral DNAs (full-length RC plus full-length SS) between WT and I97L was approximately 2.7 to 1 on day 3, but this ratio shifted to approximately 8 to 1 on week 1. In other words, the total viral DNA of I97L appeared to be 3-fold further reduced than that of the WT HBV, suggesting that the viral DNA level of I97L is relatively less persistent than the WT HBV. Furthermore, these data suggest that the lower level of mutant I97L DNA was not due to any decrease in viral RNA, capsid expression, or transfection efficiencies in hydrodynamic delivery. It is reminiscent of HBc mutant F97L, which exhibited a pleiotropic phenotype, including both a *cis*-defect in viral DNA synthesis and a *trans*-defect in virion secretion (32; see also the Discussion).

The difference in the viral DNA levels between WT and mutant I97L could originate from either the decreased synthesis or increased secretion or degradation of mutant I97L viral DNA (Fig. 2 and 3A). For easier comparison of the kinetics in the disappearance of HBV DNA between WT and I97L, we adjusted the amount of the input plasmids by using 10 μg of WT and 30 μg of I97L DNAs. This adjustment successfully generated near-equal signal intensities by Southern blotting between WT HBV and mutant I97L at 3 dpi (Fig. 3C, left panel). However, at 2 weeks postinjection (right panel), DNA signals of mutant I97L were undetectable, when a significant amount of WT viral DNA remained apparent. Consistent with these viral DNA results, the intracellular level of capsid particles of mutant I97L was significantly reduced relative to the WT HBV at 2 weeks postinjection (bottom panel). The serum HBeAg was higher for mutant I97L due to the 3-fold-higher dose of input plasmid DNA at 1 to 3 dpi (Fig. 3D). However, at 2 weeks postinjection, serum HBeAg of mutant I97L was slightly lower than that of the WT. Overall, the intracellular HBV DNA of mutant I97L indeed declined more rapidly than that of WT HBV.

### Characterization of mutant I97L in immunodeficient mice.

**FIG 4:**
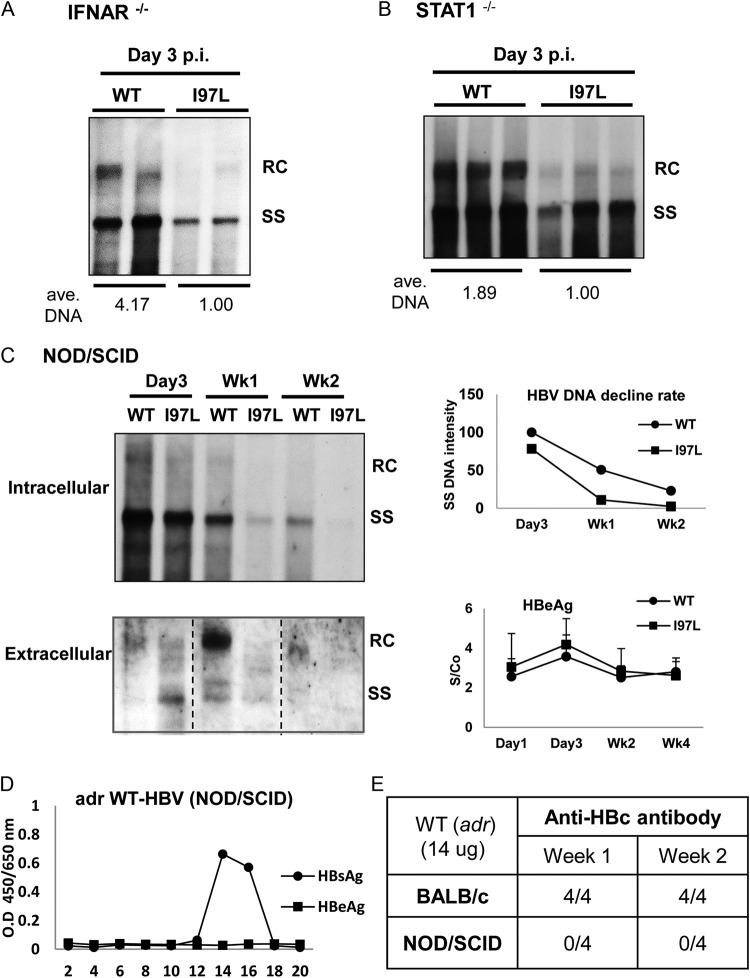
Mutant I97L replicates less efficiently than wild-type HBV in three different kinds of immunodeficient mice. (A) IFN-α receptor knockout (IFNAR^−/−^) mice were hydrodynamically injected with 30 μg of DNA of WT or mutant I97L HBV. Mouse liver was collected on 3 dpi, and HBV DNA was extracted for Southern blot analysis. Each lane represents one viral DNA sample extracted from 100 mg of the liver mass of one injected mouse. The overall viral DNA synthesis of mutant I97L is significantly reduced relative to WT HBV. The results here represent one of two independent repeat experiments. (B) STAT1^−/−^ mice were hydrodynamically injected with 30 μg of HBV plasmid DNA. The viral DNA levels of mutant I97L, particularly the RC form, were greatly reduced in the liver at 3 dpi by Southern blotting. Each lane represents one viral DNA sample extracted from 100 mg of the liver mass of one injected mouse. The averaged amounts of total viral DNA were measured and calculated as described in Fig. 2D. (C, left panel) At different time points postinjection into NOD/SCID mice, intracellular core-associated HBV DNA in the liver (upper panel) and extracellular HBV DNA in the sera (lower panel) were compared between WT HBV and mutant I97L by Southern blotting. In both panels, each lane represents pooled HBV DNA samples from three to four mice in the same experimental group. (Upper right panel) A faster decline rate of mutant I97L SS DNA was observed in a time course experiment. (Lower right panel) Similar levels of serum HBeAg were observed by ELISA in the same time course experiment. The results here represent one of two repeat experiments. (D) No naked core particles can be detected in serum samples of HBV DNA-injected NOD/SCID mice. Serum samples were subjected to cesium chloride gradient centrifugation, and HBsAg-positive fractions were detected by HBsAg ELISA (see Materials and Methods). No positive signal was detected in all fractions by HBeAg ELISA. (E) Anti-core antibody can be detected in the serum samples of hydrodynamically injected BALB/c mice but not in immunodeficient NOD/SCID mice. Anti-core antibody was measured by the ELISA (see Materials and Methods). 4/4, four of four mice were serum anti-HBc positive; 0/4, none of four mice was serum anti-HBc positive.

HBV does not induce a significant IFN response (14, 56, 57). However, hydrodynamic injection with high-dose HBV DNA (≥20 μg DNA/mouse) could induce alpha/beta interferon (IFN-α/β), which in turn suppressed HBV replication (58). In contrast, injection with a lower dose of input HBV plasmid (≤14 μg of DNA/mouse) could result in more persistent viral DNA replication. Since we used a high dose of mutant I97L plasmid DNA (30 μg of DNA/mouse) in Fig. 3C, the possibility that the lower level of mutant I97L viral DNA at 2 weeks postinjection was caused by the high-dose DNA induced innate immunity cannot be excluded. To address this issue, we performed the hydrodynamic delivery experiment using immunodeficient mouse systems (Fig. 4).

We first compared the viral DNA replications between WT HBV and mutant I97L in the livers of IFNAR^−/−^ mice at 3 days postinjection with equal amounts of plasmid DNAs (30 μg/mouse) by Southern blotting (Fig. 4A). Consistent with the experimental results using an immunocompetent mouse model (Fig. 2 and 3), mutant I97L exhibited an ∼4-fold-lower level of total intracellular viral DNAs (full-length RC plus full-length SS) than WT HBV. Next, we used STAT1^−/−^ mice defective in the signaling of IFN-α, -β, and -γ (Fig. 4B). Again, relative to WT HBV, we observed a nearly 2-fold-lower level of viral DNA of mutant I97L at 3 dpi with 30 μg of DNA per mouse. Overall, host innate immunity does not seem to play a role in the lower level of viral replication of HBc mutant I97L.

The substitution at HBc amino acid 97 of mutant I97L could represent an immune escape mutation since it coincides with potent T-cell epitopes (17). To examine the potential role of adaptive immunity in the lower level of viral DNA in mutant I97L, we extended our study to NOD/SCID immunodeficient mice, which are deficient in mature T and B cells, and with attenuated NK cells (Fig. 4C). Again, we observed lower levels of intrahepatic viral DNA of mutant I97L, relative to the WT HBV, at 3 days, 1 week, and 2 weeks postinjection (Fig. 4C, upper left panel). Because very weak or no RC DNA signals were detected at weeks 1 and 2, we compared the differences of intracellular SS DNA between WT and I97L strains. As shown in the upper right panel, Fig. 4C, the difference in viral SS DNA appeared to be further enlarged from day 3 to week 1. Similarly, in the mouse sera (Fig. 4C, lower left panel), mutant I97L secreted strong signals of immature genomes on day 3 postinjection. However, at 1 or 2 weeks postinjection, the secreted mutant I97L DNA disappeared rapidly and exhibited much lower DNA signals than did WT HBV. This result strongly supports the notion that the viral DNA associated with mutant I97L virions is less persistent than that of WT HBV at later time points postinjection. In contrast to the differential kinetics in HBV DNA persistence between WT and mutant I97L, the levels of serum HBeAg remained similar throughout the time course (Fig. 4C, lower right panel). Here, similar to the results from BALB/c mice in Fig. 2C, we detected no naked core particles in NOD/SCID mice (Fig. 4D). As expected, although anti-core antibody was detected in BALB/c mice, it was not detectable in NOD/SCID mice (Fig. 4E). Therefore, the absence of naked core particles in NOD/SCID mice is not related to any neutralization by anti-core antibody. In summary, the poor replication and persistence of viral DNA of mutant I97L were likely not due to any differential innate or adaptive immune responses to mutant versus WT HBV.

### Inefficient rescue of I97L immature secretion by an HBc mutation P130T.

**FIG 5:**
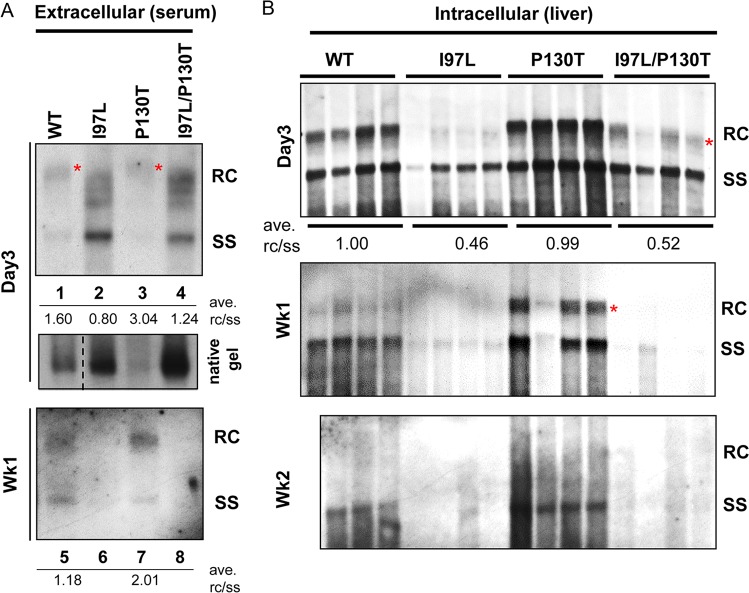
Correlation between HBV genome maturation and *in vivo* persistence. Both intracellular and extracellular viral DNAs were compared among four different groups of BALB/c mice hydrodynamically injected with 30 μg of plasmid DNAs of WT, mutant I97L, mutant P130T, and double mutant I97L/P130T, respectively. (A) Extracellular virion-associated HBV DNAs were prepared from pooled mouse sera of the same experimental group at 3 and 7 dpi, followed by Southern blotting. As highlighted by a red asterisk, fully mature full-length RC DNAs (lanes 2 and 4) in mutant I97L virions were significantly reduced. Mutation P130T appeared to partially upshift the immature SS DNA of mutant I97L to the higher-MW RC form at 3 dpi (lane 2 versus lane 4). The RC/SS ratio reflects the degree of genome maturity. Because of the smearing signals of the nascent RC replicative intermediates, quantitation of the RC DNA here was done by measuring all of the signals above the full-length SS DNA position. In the middle panel, mutation I97L resulted in higher levels of extracellular virions at 3 dpi by native agarose gel and Western blot analysis with anti-core antibody. In the bottom panel, poor persistence of I97L viral DNA was not rescued by P130T at 1 week postinjection (lane 6 versus lane 8). (B) Intracellular core-associated HBV DNA was extracted from liver tissues at 3 days, 1 week, and 2 weeks postinjection before Southern blot analysis. The characteristic hypermaturation phenotype of single mutant P130T was most pronounced at 1 week postinjection (red asterisk in the middle panel). Intracellular viral DNA of mutant P130T was also more persistent than WT and I97L mutants at 1 and 2 weeks postinjection. Mutation I97L appeared to be dominant over mutation P130T *in vivo*, since both the intracellular deficiency in RC DNA and poor persistence of mutant I97L were not significantly rescued by the mutation P130T. The results here represent one of three independent repeat experiments.

Previously, we reported another frequent, naturally occurring proline-to-threonine mutation at HBc amino acid 130 (P130T) in human hepatomas in Taiwan (17). This P130T mutant displayed a hypermaturation phenotype by accumulating more abundant amounts of full-length RC form DNA and is highly efficient in rescuing the immature secretion phenotype of mutant I97L in the human hepatoblastoma HepG2 cell line (48). In other words, an I97L/P130T double mutant in HepG2 cells behaved like WT HBV in preferential secretion of virions containing mature genome. To examine whether mutation P130T can rescue *in vivo* the immature secretion of mutant I97L, we performed hydrodynamic delivery and compared the virion secretions from four different plasmids in BALB/c mice: WT, I97L, P130T, and a double mutant I97L/P130T. The virion-associated HBV DNAs were extracted from pooled mouse sera before Southern blot analysis. As expected, the single mutant I97L secreted predominantly immature HBV genomes, including SS DNA (Fig. 5A, lane 2). Relative to that of WT HBV, at 3 day or 1 week postinjection, the single mutant P130T exhibited an extracellular DNA profile with a 2-fold-increased RC/SS DNA ratio, indicative of a hypermaturation phenotype. However, the I97L/P130T double mutant exhibited only a minor, yet reproducible effect on the upshifted viral DNA profile: from lower-MW SS DNA to higher-MW RC DNA (compare lanes 2 and 4). Unlike the cell culture system (48), immature secretion was only rescued here at a low efficiency (RC/SS ratio 0.80 versus 1.24). As was observed in Fig. 2, both mutants I97L and I97L/P130T appeared to be deficient in the fully mature, full-length RC DNA molecule (red asterisk lanes 2 and 4, Fig. 5A). At 3 days postinjection, extracellular viral DNA signals of mutants I97L and I97L/P130T were much stronger than those of WT and mutant P130T (compare lanes 1 and 3 versus lanes 2 and 4, Fig. 5A). Similar results were observed for the virions on the native agarose gel (middle panel, Fig. 5A). However, the opposite was observed at 1 week postinjection, when almost no viral DNA signal was detected in the sera from mice injected with mutants I97L or I97L/P130T (compare lanes 5 and 7 to lanes 6 and 8, Fig. 5A). Here, the more rapid decline of I97L viral DNAs is entirely consistent with the previous results from various mouse models (Fig. 3 and 4).

### Genome hypermaturation of HBc mutant P130T correlated with prolonged persistence of viral DNA.

In addition to the studies on the rescue of virion secretion (Fig. 5A), we examined whether the deficiency in the intracellular viral DNA replication of mutant I97L can be rescued by the mutation P130T (48). At 3 dpi (upper panel), similar levels of intracellular SS DNAs were observed between the single mutant I97L and the double mutant I97L/P130T. On the other hand, the RC DNA signals (*) from the double mutant I97L/P130T were slightly stronger than from the I97L single mutant (Fig. 5B, upper panel). However, the rescue efficiency of intracellular RC DNA of mutant I97L by P130T is insignificant (RC/SS ratio of 0.46 versus 0.52).

Consistent with the previously reported hypermaturation phenotype based on the HepG2 cell culture system (48), the single mutant P130T displayed a more predominant signal intensity of the fully matured RC form DNA than did WT and the other mutants at day 3 (Fig. 5B, upper panel). It is particularly striking that, at week 1, the RC DNA signal of mutant P130T was much stronger than those in the WT HBV. Above all, at 1 or 2 weeks postinjection (Fig. 5B, lower panels), the HBV DNA of mutant P130T was clearly more persistent than those of the WT, mutant I97L, and double mutant I97L/P130T strains. In summary, mutant P130T with genome hypermaturation appeared to be more persistent than WT HBV. In contrast, mutants I97L and I97L/P130T contained reduced amounts of intracellular mature genome and were less persistent than WT HBV. Mutation I97L was dominant over mutation P130T, since the latter could not successfully reverse the rapid decline of both intracellular and extracellular HBV DNA in the double mutant I97L/P130T at later time points postinjection. Interestingly, as summarized in Table 1, the prolonged persistence of intracellular viral DNA of mutant P130T is very well correlated with its phenotype of genome hypermaturation (see Discussion).

**TABLE 1:** Correlation between persistence and genome maturation

HBc variant	Genome maturity^*a*^	Persistence^*b*^
I97L	**+**	**+**
Wild type (*adr*)	**++**	**++**
P130T	**+++**	**+++**

a +, Immature genome; ++, mature genome; +++, hypermature genome.

b Intrahepatic RC DNA detectable at postinjection (+, 3 days; ++, 1 week; +++, 2 weeks).

### Full-length RC DNA generated *in vitro* by mutant I97L in an endogenous polymerase reaction.

**FIG 6:**
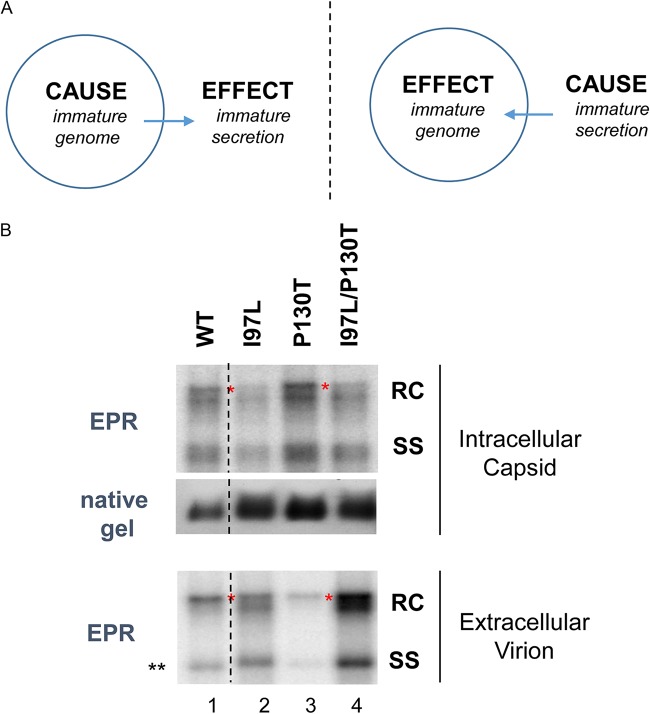
Mutant I97L can synthesize full-length RC form by *in vitro* endogenous DNA polymerase reaction (EPR). (A) Two different hypotheses for the cause-effect relationship between genome maturation and virion secretion (see Discussion for details). The left panel postulates that an intracellular defect in genome maturation is the primary cause of the extracellular immature secretion of mutant I97L. The right panel postulates that an overly efficient and less selective envelopment is the primary cause of the intracellular deficiency in the fully mature RC DNA in mutant I97L. (B) As described in the Fig. 5 legend, BALB/c mice were hydrodynamically injected with four different HBV constructs (WT, I97L, P130T, and I97L/P130T). At 3 dpi, liver lysate and pooled sera were used to purify HBV capsids for EPR as described previously (see Materials and Methods). *In vitro*-elongated viral DNAs were extracted after EPR. As an internal control, intracellular capsids were measured by native agarose gel electrophoresis, followed by Western blotting using anti-core antibody. Red asterisks highlight the fully mature full-length, double-stranded DNA of the RC form. **, the minor difference in the mobility of SS DNA between WT and I97L (lanes 1 and 2) is not reproducible, probably due to experimental variation in salt concentrations between these two particular samples. The results represent one of three independent repeat experiments.

Mutant I97L is deficient in the fully mature full-length RC DNA, as highlighted by a red asterisk in Fig. 2A and B and Fig. 5A. It remains unclear whether this phenotype could be in part due to a *cis*-defect in viral DNA synthesis (e.g., an intrinsic deficiency in making the full-length plus-strand DNA) or is entirely due to an overly efficient envelopment and export of immature capsids of mutant I97L (Fig. 6A). In the latter case (right panel), intracellular nucleocapsids of mutant I97L are exported prematurely before their DNA replicative intermediates have a chance to reach the full-length genome size. To distinguish between these two possibilities, we performed endogenous polymerase reactions using capsids prepared from four different sources of HBV: WT, I97L, P130T, and I97L/P130T. As shown in Fig. 6B, the full-length RC form (*) of both mutants I97L and I97L/P130T can be achieved by an endogenous polymerase reaction (EPR) *in vitro* by using nucleocapsids purified from intracellular lysates or extracellular virions (see Materials and Methods). This result indicated that neither intracellular nor extracellular phenotypic defects in the full-length RC form of mutant I97L are due to an inability to generate *in vitro* the full-length genome. Of note, the EPR assay did not compare kinetically the efficiencies of viral DNA synthesis between WT and I97L.

## DISCUSSION

Using a hydrodynamic delivery approach, we examined *in vivo* viral DNA synthesis and virion secretion of two naturally occurring HBc variants. As summarized in Table 2, there are similarities and differences in both DNA synthesis and virion secretion between the *in vitro* cell culture system and the *in vivo* mouse models. By immunohistochemical staining, we noted no apparent histopathological difference between liver sections from mice injected with WT HBV versus those injected with mutant I97L.

**TABLE 2:** Summary of intracellular and extracellular phenotypes of HBV core variants

HBV variant	Intracellular or extracellular	Phenotype^*a*^		Reference(s); figure(s)
		*In vitro*	*In vivo*	
Wild type (*adr*)	Intracellular	Exhibits normal RC and SS	Exhibits a normal pattern of RC and SS DNA	32, 33; Fig. 2 to 5
	Extracellular	Mature virion secretion	Mature virion secretion	32, 33; Fig. 2 to 5
				
I97L	Intracellular	WT-like in HepG2	Lower amount of RC and SS	32–34; Fig. 2 to 5
		Replication advantage in HuH-7	Decreased fully mature full-length RC DNA	32–34; Fig. 2 and 5
			Less persistent	32–34; Fig. 3 to 5
	Extracellular	Immature secretion	Immature secretion	32, 33; Fig. 2, 4, and 5
			Excessive virion secretion (3 dpi)	32, 33; Fig. 2, 4, and 5
			Less persistent	32, 33; Fig. 2, 4, and 5
				
P130T	Intracellular	A hypermaturation phenotype^*b*^	Hypermaturation	48; Fig. 5
			More persistent	48; Fig. 5
	Extracellular		Weak hypermaturation	48; Fig. 5
				
I97L/P130T	Intracellular	WT-like RC and SS	Partial rescue of RC DNA	48; Fig. 5
			Less persistent	48; Fig. 5
	Extracellular	Complete rescue of immature secretion	Partial rescue of immature secretion	48; Fig. 5

a *In vitro*, cell culture; *in vivo*, mouse model.

b A hypermaturation phenotype is characterized by enhanced signal of fully mature full-length RC DNA (Fig. 1).

The most prominent feature of mutant I97L is the relaxed stringency in the release of virions containing immature genomes of low-MW RC and SS DNA. This immature secretion phenotype can be faithfully recapitulated in mice (Fig. 2A and B), irrespective of their immunogenetic backgrounds (Fig. 4C). Another novel finding in the time course experiments was the poor persistence of the intracellular and extracellular viral DNAs of mutant I97L relative to the WT HBV (Fig. 3 and 4). Even though the intracellular mutant I97L DNA was even stronger in signal intensity than WT DNA at day 3 postinjection, no viral DNA from mutant I97L was detected at 2 weeks postinjection, when the WT DNA remained clearly detectable (Fig. 3C). A lower level of mutant I97L DNA was also observed in the extracellular virions at 1 or 2 weeks postinjection in both immunocompetent BALB/c and immunodeficient NOD/SCID mouse models (Fig. 4C and Fig. 5A). Because NOD/SCID mice are deficient in mature T and B cells, more rapid clearance of mutant I97L virions could not be related to the humoral immunity. Similarly, more rapid decay of the intracellular DNA of mutant I97L may not be related to the innate immunity, since the same phenomenon was observed in immunocompetent BALB/c mice and immunodeficient IFNAR^−/−^ or STAT1^−/−^ mice (Fig. 3C and Fig. 4A and B). If host immunity is not the primary mechanism for poor persistence of mutant I97L, what could then be the mechanism behind the phenomenon of poor persistence of mutant I97L? The most apparent clue for the mechanism of poor persistence is its correlation with the genome immaturity of the extracellular HBV DNA.

We entertain here two different hypotheses for the immature secretion phenotype. (i) In the first hypothesis (Fig. 6A, left panel), HBc mutant 97L could have an intrinsic problem in DNA synthesis due to a subtly altered capsid structure. For example, in a *cis-trans* complementation experiment (32, 36), a wild-type core protein was provided in *trans* to two different replicon plasmids in two separate cotransfections side by side. One plasmid contains only a single mutation in HBc protein translational initiation codon AUG. The other plasmid contains double mutations, with an additional F97L mutation. Despite the fact that the same wild-type core protein was provided to these two different replicons, Southern blot analysis revealed that the double mutation replicon exhibited a 2.4-fold reduction in minus-strand DNA synthesis, in addition to a 10-fold reduction in plus-strand DNA synthesis (32, 36). Therefore, mutation F97L is pleiotropic in both *cis*- and *trans*-defects in cell culture. Here, we have not performed the same *cis-trans* test *in vivo* for mutant I97L. In fact, in HepG2 cells, we detected no apparent deficiency in the intracellular DNA synthesis of mutant I97L (33). The EPR result alone cannot exclude the first hypothesis, since it did not measure the efficiency or kinetics in DNA synthesis. (ii) In the second hypothesis (Fig. 6A, right panel), a superefficient interaction between the viral envelope protein and the nucleocapsid particles could be responsible for the intra- and extracellular phenotypes of mutant I97L. Once envelopment and virion secretion occur, genome maturation is then arrested. Previously, we demonstrated that a mutation A119F in the pre-S1 domain of the envelope protein can rescue the immature secretion phenotype of mutant I97L (51). This study suggests that virion secretion is most likely to involve the interaction between the pre-S1 domain and the nucleocapsids. Indeed, we noted that the stronger intensity of the extracellular virion-associated DNA of mutant I97L (Fig. 2A and B) is correlated with its reduced intensity of the intracellular capsid-associated DNA (Fig. 2D). This inverse correlation suggests that mutant I97L is more efficient in virion secretion than WT HBV, leading to the depletion of the intracellular pool of viral DNA, including the RC and SS DNAs. Indeed, in our previous study, when virion secretion of mutant F97L was genetically blocked with another surface antigen knockout mutation (SK/O), intracellular genome maturation to full-length RC DNA was well restored (∼30%) in the double mutant F97L/SK/O (32). Therefore, the *cis*-defect in viral DNA synthesis of mutant F97L contributes to approximately 70% of the reduction in the total intracellular viral DNA. Similarly, we speculate here that HBc mutation I97L could generate a mutant nucleocapsid with a subtly altered structure (52–54), leading to pleiotropic phenotypes, including a relaxed stringency in envelopment for immature nucleocapsids, as well as a *cis*-defect in viral DNA synthesis.

Previously, we reported a naturally occurring mutation, P130T, which displayed a hypermaturation phenotype with more abundant amount of the fully mature RC form-DNA than did WT HBV in cell culture (48). This mutation P130T can efficiently rescue the immature secretion of mutation I97L in the cell culture system. In an *in vivo* experimental setting, mutant P130T still exhibited more highly abundant fully mature RC-form DNA than did the WT HBV (Fig. 5B, middle panel). However, unlike the cell culture system (48), the immature secretion phenotype from mutant I97L could only be partially rescued by P130T in the double mutant I97L/P130T (only partially upshifted RC DNA in the day 3 panel) (Fig. 5A).

An unexpected result shown in Fig. 5B is that, relative to WT HBV, there was a more prolonged persistence of HBV DNA of the hypermaturation mutant P130T in the mouse liver at 1 and 2 weeks postinjection. Conversely, also in Fig. 5B, mutant I97L deficient in the intracellular mature genome was less persistent than was WT HBV. Therefore, there is an apparent correlation between HBV genome maturation and the persistence of intracellular HBV DNA *in vivo*. It is tempting to speculate here that the mature RC DNA in the nucleocapsids of P130T might shuttle back to the nucleus for cccDNA amplification (11, 59). As such, the fully mature RC DNA could serve as a precursor to cccDNA and thus could play a more important role in the persistence of viral DNA than undergoing envelopment and secretion (1, 2). This is particularly true for our current hydrodynamic mouse model, which can support HBV replication and virion secretion, but not infection or reinfection. Previously, cccDNA was not detected by Southern blotting in the livers of transgenic or hydrodynamically injected mice (60, 61). On the other hand, when HBV transgenic mice were crossed to HNF1-α knockout mice, cccDNA was detected in the mouse nuclei by Southern blotting (62, 63). It is possible that the formation of cccDNA can still occur in hydrodynamically injected mouse hepatocytes; however, unlike human hepatocytes, cccDNA can only exist transiently in the mouse liver due to its high instability.

It was reported that HBc of mutant F97L exhibited faster kinetics in empty capsid assembly (64). Compared to WT HBc, mutant F97L showed an enhanced rate and extent and a stronger temperature dependence of empty capsid assembly *in vitro*. It remains to be investigated whether these *in vitro* capsid assembly properties of mutant F97L could also influence the efficiency of envelopment in virion secretion in mouse models. Comparison of the secretion kinetics of mature virions in cell culture revealed no detectable difference between mutant F97L and WT HBV (51). Similarly, mutant F97L exhibited no differential secretion kinetics between its mature and immature virions (51). Finally, mutation F97L also does not have any apparent effect on HBc phosphorylation in Escherichia coli coexpressing the SRPK1 kinase (65).

Due to the difficulty in physical separation of immature from mature virions, it remains to be addressed in the future whether these immature virions are less or more infectious *in vitro* than *in vivo*. If less infectious *in vivo*, then how can mutant I97L emerge to predominance in chronic hepatitis B patients in the first place (15, 16, 66)? In longitudinal studies, putative immune-selected HBc variants can emerge after a single or repeated acute exacerbations (67, 68). Given the fact that the phenomenon of immature secretion can be observed in both cell culture and hydrodynamic mouse models, it is natural to ask whether immature secretion can be detected in chronic patients. Indeed, immature genomes of low-MW HBV DNA can be detected by Southern blotting using serum HBV samples containing a predominant HBc mutation I97L (34). However, there are two caveats or complications here. One is the sequence heterogeneity in HBV population in the individual patient’s serum. The other complication is that the HBV DNA from human patients always contains multiple mutations in addition to I97L or F97L (e.g., coexisting with known or unknown compensatory mutations) (34). It is therefore difficult to correlate the virion-associated immature genomes in the serum with any particular hot spot mutation in HBV DNA sequences.

As mentioned earlier, a hydrophobic pocket in the center of the spikes around amino acid 97 was shown to be important for both DNA synthesis and virion secretion (52). Significant structural differences in this hydrophobic pocket were revealed using cryoEM analysis by comparing mature versus immature capsids or virions (53, 54). Upon systematic substitution at HBc amino acid 97 from a wild-type isoleucine to 18 other amino acids via site-directed mutagenesis, only the mutant I97L exhibited immature secretion (52). These earlier studies led to the hypothesis that a hydrophobic pocket around amino acid 97 could be involved in the signal transduction of genome maturation. This *in vivo* platform could facilitate further studies on the molecular mechanism of HBV virion secretion. To our knowledge, this is the first *in vivo* demonstration of an immature secretion phenotype of a frequent naturally occurring HBc variant I97L in a mouse model. Most surprising here is the finding that HBV genome maturation appears to be very important for the persistence of intracellular HBV DNA in hepatocytes. This finding provides a plausible rationale for the WT hepadnavirus to couple its secretion of virions with the mature genome (25) instead of the immature genome (32–34).

## MATERIALS AND METHODS

### Ethics statement.

All animal experiments were conducted under protocols approved by the Academia Sinica Institutional Animal Care and Utilization Committee (ASIACUC permit 12-02-322). Research was conducted in compliance with the principles stated in the *Guide for the Care and Use of Laboratory Animals* (National Research Council, 1996).

### Hydrodynamics-based transfection.

BALB/c mice were purchased from the National Laboratory Animal Center (Taipei, Taiwan). NOD/SCID mice were purchased from BioLASCO (Taiwan). IFNAR^−/−^ and STAT1^−/−^ mice were as described elsewhere (69–71). The mice were fed with standard chow and water *ad libitum*. Six- to eight-week-old male mice were anesthetized with ketamine and xylazine. Mice were coinjected via tail vein with 10 to 30 μg of HBV *adr* tandem dimer and 2.5 μg of secretory alkaline phosphatase (SEAP) in normal saline in a volume equivalent to 8 to 10% body weight.

### Southern and Northern blot analysis.

HBV core-associated DNA from transfected liver tissues were subjected to Southern blotting as previously described (11) using a digoxigenin-labeled HBV DNA probe (nucleotides 1521 to 3164, *ayw* numbering system; Roche). HBV viral RNA was prepared by using TRIzol reagent and detected by Northern blotting of total liver RNA using a random-primed ^32^P-labeled DNA probe (Amersham Rediprime II DNA labeling system; GE). Band intensities were quantified by using ImageJ software.

### Detection of serum HBV antigen and secreted alkaline phosphatase.

The serum levels of HBeAg, HBsAg, and anti-HBc antibody of mice were determined by using an ELISA kit (General Biologicals Corporation, Taiwan) according to the manufacturer’s protocols. Positivity for HBeAg and HBsAg was determined from signal/cutoff ratios (S/Co) of ≥1. Anti-HBc positivity is determined by a S/Co of <1. Serum levels of SEAP were determined by using SIGMA*FAST p*-nitrophenyl phosphate tablets (Sigma) according to the vendor’s protocols.

### Analysis of virion secretion and CsCl density gradient centrifugation.

Mouse sera from the same experimental group were pooled before loading onto the 20% sucrose cushion. The CsCl gradient (20 to 50% [wt/vol]) for centrifugation analysis of secreted viral particles was as detailed elsewhere (21, 22).

### Endogenous polymerase reaction.

Purified HBV capsids from liver and sera were used to perform the endogenous DNA polymerase reaction (72). Briefly, liver lysates were first immunoprecipitated with rabbit anti-HBc antibody to isolate HBV capsids. Membrane and envelope proteins were removed from virions in the pooled serum samples by treatment with 1% NP-40 at 37°C for 30 min. Membrane-free capsids were subjected to 20% sucrose cushion centrifugation. Precipitated HBV capsids at the bottom of the tube were resuspended in a buffer (10 mM Tris-HCl, 50 mM NaCl, 10 mM MgCl_2_, and 1 mM dithiothreitol [pH 7.9]) before adding 25 mM dATP, 25 mM dGTP, 25 mM dTTP, and 10 μCi [α-^32^P]dCTP. The EPR reaction mixture was incubated for 2 h at 37°C. Cold 25 mM dCTP was added to the reaction for 1 h in 37°C. For extraction of *in vitro*-elongated viral DNAs, EPR samples were treated with 0.3 mg/ml protease K and 1% sodium dodecyl sulfate (SDS), followed by standard phenol-chloroform extraction and ethanol precipitation. Extracted viral DNA samples were subjected to agarose gel electrophoresis, and the images were scanned with a Typhoon 9410 model imager (Amersham BioScience, Piscataway, NJ).

### HBV replicon plasmid.

The HBV (*adr*) plasmid is described elsewhere (73; GenBank accession number AY123041).

### Native agarose gel and Western blotting of core particles.

The purification of core particles from serum and liver samples were as described previously (9, 55). The transfer buffer of Western blot contains 0.1% SDS.
